# Identification of Key Circulating Exosomal microRNAs in Gastric Cancer

**DOI:** 10.3389/fonc.2021.693360

**Published:** 2021-07-16

**Authors:** Xiaoqing Qian, Feng Xie, Huabing Wei, Daxiang Cui

**Affiliations:** ^1^ School of Biomedical Engineering, Shanghai Jiaotong University, Shanghai, China; ^2^ Department of Instrument Science & Engineering, School of Electronic Information & Electrical Engineering, Shanghai Engineering Research Center for Intelligent Diagnosis & Treatment Instrument, Institute of Nano Biomedicine & Engineering, Shanghai Jiaotong University, Shanghai, China; ^3^ Department of thoracic Surgery, Renji Hospital, School of Medicine, Shanghai Jiaotong University, Shanghai, China

**Keywords:** gastric cancer, exosome, miRNA, plasma, bioinformatics analysis

## Abstract

Exosomal miRNAs (EmiRs) can be used for prediction of gastric cancer (GC) development. Supposedly, both plasma and urinary microRNAs can also be potential biomarkers for screening, but the diagnostic values of EmiRs in blood and urine are not fully studied. We here collected both types of samples from GC patients and healthy individuals and conducted miRNA sequencing to identify key members of EmiRs in GC. The exosomes samples derived from blood and urine were collected from 3 healthy individuals and 7 GC patients. Differentially expressed miRNAs (DEmiRNAs) were acquired, ontology enrichment analysis and Protein-protein Interaction (PPI) enrichment analysis were performed. There were 8 DEmiRNAs in the serum and 3 DEmiRNAs in the urine. For GC patients, there were three up-regulated DEmiRNAs (hsa-miR-130b-3p, hsa-miR-151a-3p and hsa-miR-15b-3p) in the serum exosomes, and one up-regulated DEmiRNA (hsa-miR-1246) in the urinary exosomes. Using miRNA target prediction databases, we found 418 common targets of hsa-miR-15b-3p, 35 common targets of hsa-miR-151a-3p, 117 common targets of hsa-miR-130b-3p, and 357 common targets of hsa-miR-1246. Some commonly enriched ontology terms were found, including GO BP terms like cell surface receptor signaling pathway involved in cell-cell signaling, positive regulation of catabolic process, morphogenesis of an epithelium, and GO CC terms perinuclear region of cytoplasm. The PPI network show some key nodes, including TAOK1, CMTM6, SCN3A, WASF3, IGF1, CNOT7, GABRG1, PRKD1. Together, this study provided an integrative analysis of expression profile of key circulating exosomal microRNAs. Four key exosomal miRNAs (hsa-miR-130b-3p, hsa-miR-151a-3p and hsa-miR-15b-3p) and the interaction network or enrichments based on their targets (TAOK1, CMTM6, SCN3A, WASF3, IGF1, CNOT7, GABRG1, PRKD1) may provide a reference of the molecular mechanisms in the GC development.

## Introduction

Gastric cancer (GC) is the fourth most common malignance worldwide (1). An exact detection based on GC biomarkers has a high clinical significance. For GC patients, the exosomal microRNA (miRNA or miR) may have potential clinical application value in diagnosis and status evaluation (2, 3). MicroRNA is a short-chain noncoding RNA molecule (around 22 nucleotides in length). It regulates protein expression of a particular mRNA (by incomplete base pairing), causing inhibition of protein translation of its target genes. Exosomes are a subset of extracellular vesicles containing a variety of bioactive molecules including proteins, lipids, and RNAs. It can play a role in intercellular crosstalk. During tumor development, exosomes can facilitate construction of the specific microenvironment; besides, it strongly affects the occurrence, metastasis, and even drug resistance. Tumor secreted exosomes are sharply different among cancers, and between healthy individuals vs. cancer patients. Theoretically, the contents of exosomes include different tumor-related biomarkers. Commonly, previous studies observed the samples of plasma exosomal miRNAs (EmiRs) for prediction of GC development, progression, and treatment outcomes. Although the development of new biomarkers in blood tests has shown great potential, limited reports have applied multi-biomarkers based on the strategy of exosomal miRNA detection. Supposedly, urinary microRNA can also be a potential biomarker for tumor screening. For example, a lower urine level of miR-30a-5p was found in gastric cancer and colon carcinoma patients when compared to ovarian serous adenocarcinoma, and urinary miR-30a-5p from ovarian cancer patients was notably reduced following the surgical removal of ovarian serous adenocarcinoma (4). However, the diagnostic value of EmiRs in urine for GC is unknown, and that of plasma EmiRs is also not fully studied. We here collected both plasma samples and urine samples from GC patients and healthy control individuals for miRNA sequencing, and some key members were identified.

## Methods

### Exosomal miRNA Sequencing

The exosomes samples derived from blood and urine were collected from 3 healthy individuals and 7 GC patients (Stage I-II), including 6 blood samples (3 GC *vs* 3 controls) and 10 urine samples (7 GC *vs* 3 controls). The median age of the 7 patients was 52 years, and that of the healthy controls was 45 years. All GC patients were confirmed by histopathology. The frozen samples were thawed and centrifuged at 2000 g for 30 min to remove cells/debris. Next, 0.2 volumes of the Total Exosome Isolation reagent (Thermo Fisher, Inc.) were added, the solution was thoroughly mixed and incubated for 30 min at 4°C. Next, the samples were centrifuged at 10000 g for 10 min and the exosome pellet was resuspended in PBS. Next, the exosome-derived RNA was extracted from exosomes using the Total Exosome RNA and Protein Isolation Kit, the total amount of RNA was detected. Subsequently, the Illumina NextSeq 500 SE50 (20M) sequencing was performed. After sequencing, the FastQ-format data were stored and cleaned for further analysis.

### Differential miRNAs Analysis

First, miRNA expression was analyzed by miRExpress (http://mirexpress.mbc.nctu.edu.tw), which works based on the miRNA database (http://www.mirbase.org) for quantitative analysis of miRNA. After the second-generation sequencing raw data in FastQ format are imported, the software compares the data with the known miRNA sequence in miRBase and then acquires the read count of the same sequence fragments as Counts, which is used to measure the miRNA levels in a sample. According to the grouping information, the sample expression data was imported into the edgeR R package to compare the expression differences between samples. The Fold Change and p-value were obtained. Then, according to the threshold of p-value<0.05 and Log(Foldchange) >2 or <-2, the DEmiRNAs was acquired. The volcanic plots were produced to present DEmiRNAs using the ggplot2 R package. And a principal component analysis (PCA) was performed using the ggfortify R package based on all detected miRNA levels.

### Expression Analysis of DEmiRNAs in TCGA and EVmiRNA

Exosomal miRNAs that are up-regulated in the urine or blood exosomes of tumor patients have potential clinical applications, so the differential miRNAs obtained from screening were subjected to expression analysis. From the Cancer Genome Atlas (https://portal.gdc.cancer.gov/) and EVmiRNA (http://bioinfo.life.hust.edu.cn/EVmiRNA#!/) databases, the raw count of tumor and exosomal miRNA expression data were obtained. And box plots were drawn by ggplot2 R software.

### Prognostic Analysis of DEmiRNAs in TCGA

Corresponding clinical information were obtained from The TCGA dataset. High and low expression of miRNAs was defined with the median. For the Kaplan-Meier curve, the p value and the hazard ratio (HR) with 95% confidence interval (CI) are obtained by logrank test and univariate Cox proportional hazard regression. p<0.05 was considered statistically significant. And p < 0.05 was considered as statistically significant.

### Target Prediction of the DEmiRNAs

First, we focused on the up-regulated DEmiRNAs which may be encapsulated in circulating exosomes. The mRNAs potentially targeted by these miRNAs were predicted using both miRDB and Targetscan databases. When the intersection of two databases regarding the targets of each up-regulated DE-miR was acquired, the overlap set of mRNAs were further used for enrichment analysis.

### Predicted Target Gene Enrichment Analysis

The Metascape gene list analysis online tool was used for enrichment analysis of the predicted target genes. All targets were imported into the tool, gene annotation was performed, and pathway and process enrichment analysis were carried out with the following ontology sources: KEGG Pathway, GO Biological Process, GO Molecular Function and GO Cell Component. The Homo Sapiens background was used for enrichment analysis of all predicted target genes. Terms with a p-value < 0.01, a minimum count of 3, and an enrichment factor > 1.5 (the ratio between the observed counts and the counts expected by chance) were collected and grouped into clusters based on their according DEmiRNAs. The statistically enriched terms were presented by accumulative hypergeometric p-values and enrichment factors, and then hierarchically clustered into a tree based on Kappa-statistical similarities.

### Protein-Protein Interaction Analysis

Protein-protein interaction analysis was carried out with STRING and BioGrid. And the Molecular Complex Detection (MCODE) was applied to identify densely connected network components. The MCODE networks identified for individual gene lists were gathered and drawn by the Metascape (https://metascape.org/).

### Network Hub Gene Expression Analysis

For the analysis of hub genes in the resulting MCODE subnetwork, GEPIA (http://gepia.cancer-pku.cn/) tools were used to analyze their expression profiles in the STAD from TCGA. And p < 0.05 was considered as statistically significant.

## Results

### Identification of GC Associated DEmiRNAs

**Figure 1:**
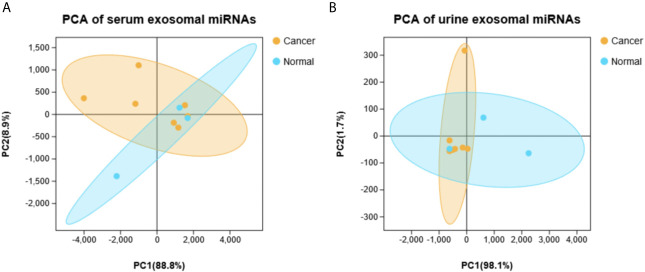
The principal component analysis (PCA) based on all detected miRNA levels. **(A)** Serum. **(B)** Urine.

**Figure 2:**
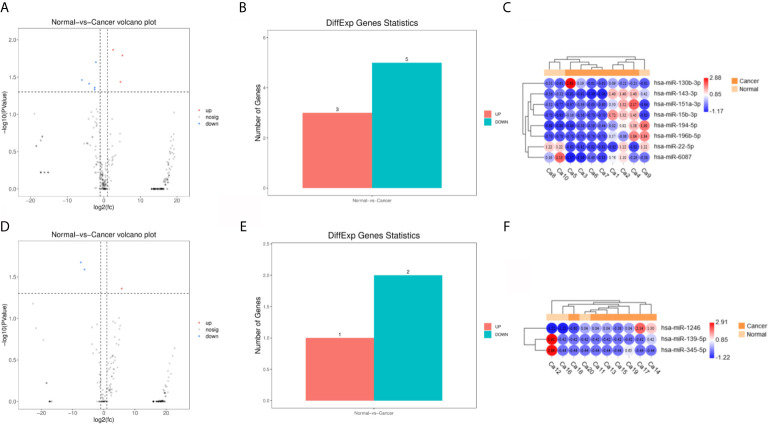
Differentially expressed miRNA (DEmiRNAs) in plasma and urine samples of the gastric cancer (GC) patients. **(A)** The volcano plots of DEmiRNAs in serum. **(B)** Significant ups and downs in serum. **(C)** Expression heatmap of significantly expressed miRNAs in serum. **(D–F)** Urine.

Principal component analysis were presented in **Figure 1**, which suggested that there is some heterogeneity in the expression profiles of blood and urine exosomal miRNAs in patients. As **Table 1** and **Figure 2** shown, there were 8 DEmiRNAs in the serum and 3 DEmiRNAs in the urine. The expression profiles of all differentially expressed miRNAs are shown in the Heatmap. For GC patients, there were 3 up-regulated DEmiRNAs (hsa-miR-130b-3p, hsa-miR-151a-3p and hsa-miR-15b-3p) in the serum exosomes, and one DEmiRNA (hsa-miR-1246) enriched in the urinary exosomes. Theoretically, these up-regulated miRNAs may be transferred to tissues or cells and target specific RNAs, which finally promotes the GC development.

**Table 1:** Differentially expressed exosomal microRNAs identified by next generation sequencing.

miRNA Information			Statistics & Regulation		
Source	Mature-miRNA	sequence	Fold Change	*P*-value	Regulation
Serum	hsa-miR-130b-3p	CAGUGCAAUGAUGAAAGGGCAU	4.555389982	0.036615174	Up
Serum	hsa-miR-143-3p	UGAGAUGAAGCACUGUAGCUC	-2.481915254	0.043638531	Down
Serum	hsa-miR-151a-3p	CUAGACUGAAGCUCCUUGAGG	2.545806244	0.013604136	Up
Serum	hsa-miR-15b-3p	CGAAUCAUUAUUUGCUGCUCUA	5.095125215	0.016185555	Up
Serum	hsa-miR-194-5p	UGUAACAGCAACUCCAUGUGGA	-2.508236592	0.046425407	Down
Serum	hsa-miR-196b-5p	UAGGUAGUUUCCUGUUGUUGGG	-4.002942023	0.038692865	Down
Serum	hsa-miR-22-5p	AGUUCUUCAGUGGCAAGCUUUA	-5.970168823	0.034517425	Down
Serum	hsa-miR-6087	UGAGGCGGGGGGGCGAGC	-2.154113907	0.019948851	Down
Urine	hsa-miR-1246	AAUGGAUUUUUGGAGCAGG	5.747077844	0.043731253	Up
Urine	hsa-miR-139-5p	UCUACAGUGCACGUGUCUCCAGU	-6.184723327	0.025724708	Down
Urine	hsa-miR-345-5p	GCUGACUCCUAGUCCAGGGCUC	-7.376130587	0.021140159	Down

Fold change, Fold change between two groups; P-value, P-value is calculated by paired t-test; Regulation, ‘up’ indicates up-regulation, and ‘down’ indicates down-regulation.

### Expression of DEmiRNAs in TCGA and EVmiRNA

**Figure 3:**
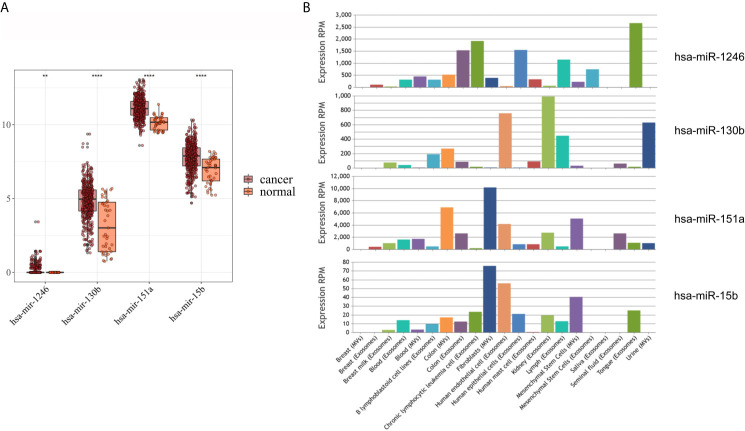
Expression analysis of DEmiRNAs in TCGA and EVmiRNA. **(A)** The expression level of DEmiRNAs in TCGA dataset. **(B)** EVmiRNA dataset.

As shown in **Figure 3A**, hsa-mir-130b-3p, hsa-mir-151a-3p and hsa-mir-15b-3p, which were significantly upregulated in serum exosomes from GC patients, were expressed at lower levels in gastric cancer tissues compared with adjacent noncancerous tissues, and hsa-mir-1246, which was significantly upregulated in urine exosomes, was barely expressed in gastric cancer tissues or adjacent noncancerous tissues. While in the EVmiRNA database, which exclusively exhibits exosomal or microvesicular miRNA expression data, the expression profiles of these four miRNAs in different tissues of origin are shown in **Figure 3B**, it is worth noting that hsa-mir-1246 has never been previously reported in urine exosomes.

### Prognostic of DEmiRNAs in TCGA

**Figure 4:**
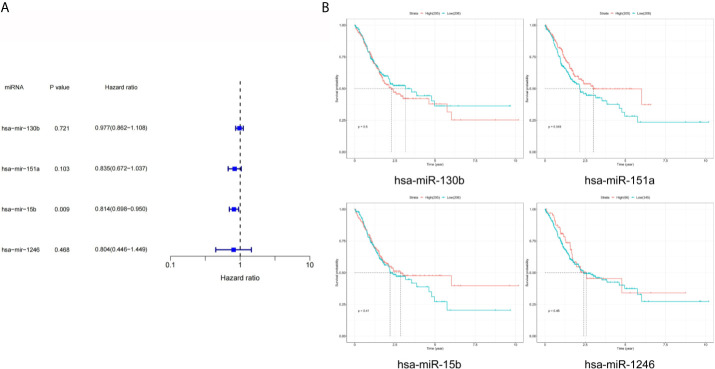
Prognostic analysis of DEmiRNAs in TCGA. **(A)** The p value, risk coefficient HR and confidence interval of the DEmiRNAs. **(B)** Kaplan-Meier survival analysis of the DEmiRNAs signature.

As shown in **Figure 4A**, the results of univariate Cox analysis regarding these four miRNAs revealed that only one serum exosomal hsa-mir-15b-3p was identified to be associated with GC patient prognosis (HR = 0.814, P < 0.05). None of the other three were related, hsa-mir-130b-3p (HR = 0.977, P > 0.05), hsa-mir-151a-3p (HR = 0.835, P > 0.05), hsa-mir-1246 (HR = 0.804, P > 0.05). While the results of prognostic analysis in **Figure 4B** were different, high hsa-mir-151a-3p expression was considered to be associated with poor survival outcomes in GC patients (P < 0.05), while the other three miRNAs were not.

### Predicted Target Gene of the DEmiRNAs

**Figure 5:**
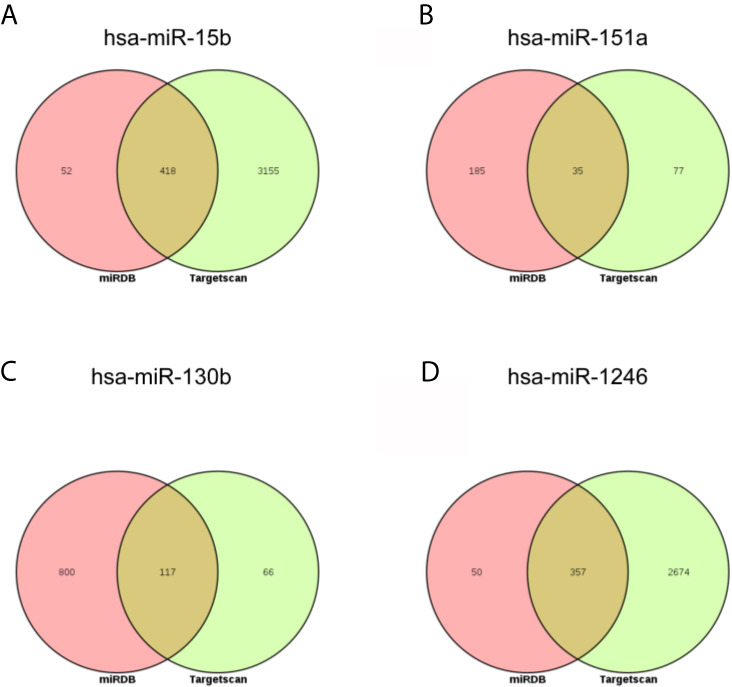
The intersection analysis of predicted target genes of DEmiRNAs. **(A)** hsa-miR-15b-3p. **(B)** hsa-miR-151a-3p. **(C)** hsa-miR-130b-3p. **(D)** hsa-miR-1246.

Next, the targets of above three miRNAs were acquired. In the miRDB database, there were 470 targets of hsa-miR-15b-3p, 220 targets of hsa-miR-151a-3p, 917 targets of hsa-miR-130b-3p, and 407 targets of hsa-miR-1246; in the Targetscan database, there were 3573 targets of hsa-miR-15b-3p, 112 targets of hsa-miR-151a-3p, 183 targets of hsa-miR-130b-3p, and 3031 targets of hsa-miR-1246. The intersect of two databases were calculated and presented in **Figure 5**. Between two databases, there were 418 common targets of hsa-miR-15b-3p, 35 common targets of hsa-miR-151a-3p, 117 common targets of hsa-miR-130b-3p, and 357 common targets of hsa-miR-1246.

### Predicted Target Gene Enrichment Analysis

**Figure 6:**
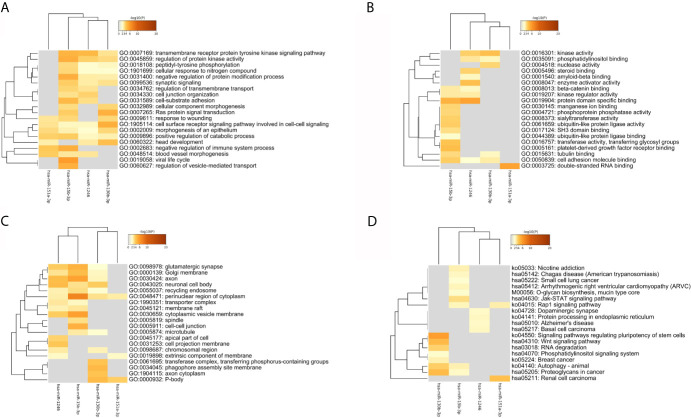
The heatmap of the enriched GO terms and KEGG pathways based all the common targets of the four key DEmiRNAs. **(A)** Biology Process. **(B)** Molecule Function. **(C)** Cellular Components. **(D)** KEGG pathway.

As **Figure 6** and **Table 2** shown, the enrichment analysis was conducted based on above targets. Some commonly enriched ontology terms were found, including GO BP terms like cell surface receptor signaling pathway involved in cell-cell signaling, positive regulation of catabolic process, morphogenesis of an epithelium, and GO CC terms perinuclear region of cytoplasm.

**Table 2:** List of enriched terms.

GO	Category	Description	Count	%	Log10(P)	Log10(q)
GO:0045859	GO Biological Processes	regulation of protein kinase activity	63	7.13	-10.64	-6.45
GO:0007169	GO Biological Processes	transmembrane receptor protein tyrosine kinase signaling pathway	59	6.68	-10.17	-6.45
GO:0031400	GO Biological Processes	negative regulation of protein modification process	48	5.44	-9.51	-5.92
GO:1905114	GO Biological Processes	cell surface receptor signaling pathway involved in cell-cell signaling	50	5.66	-9.01	-5.59
GO:0009896	GO Biological Processes	positive regulation of catabolic process	41	4.64	-8.86	-5.56
GO:0099536	GO Biological Processes	synaptic signaling	53	6	-8.09	-5.04
GO:0007265	GO Biological Processes	Ras protein signal transduction	33	3.74	-7.98	-4.96
GO:0002009	GO Biological Processes	morphogenesis of an epithelium	42	4.76	-7.19	-4.32
GO:0019058	GO Biological Processes	viral life cycle	32	3.62	-7.14	-4.29
GO:0032989	GO Biological Processes	cellular component morphogenesis	53	6	-7.11	-4.28
GO:0031589	GO Biological Processes	cell-substrate adhesion	32	3.62	-6.83	-4.04
GO:0048514	GO Biological Processes	blood vessel morphogenesis	48	5.44	-6.79	-4.02
GO:0009611	GO Biological Processes	response to wounding	47	5.32	-6.77	-4.02
GO:0034330	GO Biological Processes	cell junction organization	49	5.55	-6.75	-4.02
GO:0060627	GO Biological Processes	regulation of vesicle-mediated transport	40	4.53	-6.71	-4.02
GO:1901699	GO Biological Processes	cellular response to nitrogen compound	49	5.55	-6.71	-4.02
GO:0002683	GO Biological Processes	negative regulation of immune system process	34	3.85	-6.68	-4
GO:0060322	GO Biological Processes	head development	51	5.78	-6.61	-3.96
GO:0034762	GO Biological Processes	regulation of transmembrane transport	42	4.76	-6.57	-3.95
GO:0018108	GO Biological Processes	peptidyl-tyrosine phosphorylation	32	3.62	-6.57	-3.95
						
GO:0019904	GO Molecular Functions	protein domain specific binding	58	6.57	-10.91	-7.24
GO:0016301	GO Molecular Functions	kinase activity	50	5.66	-6.36	-3.17
GO:0050839	GO Molecular Functions	cell adhesion molecule binding	39	4.42	-5.82	-2.75
GO:0019207	GO Molecular Functions	kinase regulator activity	22	2.49	-5.73	-2.75
GO:0003725	GO Molecular Functions	double-stranded RNA binding	4	11.43	-5.6	-2.16
GO:0015631	GO Molecular Functions	tubulin binding	29	3.28	-5.16	-2.34
GO:0017124	GO Molecular Functions	SH3 domain binding	14	1.59	-4.29	-1.56
GO:0008047	GO Molecular Functions	enzyme activator activity	35	3.96	-4.28	-1.56
GO:0005161	GO Molecular Functions	platelet-derived growth factor receptor binding	4	0.96	-4.24	-1.43
GO:0016757	GO Molecular Functions	transferase activity, transferring glycosyl groups	22	2.49	-4.04	-1.41
GO:0035091	GO Molecular Functions	phosphatidylinositol binding	20	2.27	-3.8	-1.36
GO:0061659	GO Molecular Functions	ubiquitin-like protein ligase activity	14	3.35	-3.68	-1.19
GO:0008373	GO Molecular Functions	sialyltransferase activity	4	0.96	-3.63	-1.19
GO:0005496	GO Molecular Functions	steroid binding	7	1.97	-3.55	-1.15
GO:0030145	GO Molecular Functions	manganese ion binding	6	1.44	-3.5	-1.14
GO:0004518	GO Molecular Functions	nuclease activity	6	5.13	-3.49	-1.14
GO:0008013	GO Molecular Functions	beta-catenin binding	10	1.13	-3.44	-1.05
GO:0044389	GO Molecular Functions	ubiquitin-like protein ligase binding	22	2.49	-3.42	-1.05
GO:0004721	GO Molecular Functions	phosphoprotein phosphatase activity	10	2.39	-3.38	-1.09
GO:0001540	GO Molecular Functions	amyloid-beta binding	10	1.13	-3.36	-1.01
GO:0048471	GO Cellular Components	perinuclear region of cytoplasm	62	7.02	-12.08	-8.8
GO:0098978	GO Cellular Components	glutamatergic synapse	36	4.08	-10.86	-8.09
GO:0043025	GO Cellular Components	neuronal cell body	46	5.21	-10.83	-8.09
GO:0030424	GO Cellular Components	axon	54	6.12	-10.77	-8.09
GO:0000139	GO Cellular Components	Golgi membrane	56	6.34	-8.15	-5.73
GO:0030659	GO Cellular Components	cytoplasmic vesicle membrane	55	6.23	-7.67	-5.39
GO:0055037	GO Cellular Components	recycling endosome	20	2.27	-5.57	-3.61
GO:1990351	GO Cellular Components	transporter complex	28	3.17	-5.46	-3.53
GO:0045177	GO Cellular Components	apical part of cell	31	3.51	-5.03	-3.15
GO:0005911	GO Cellular Components	cell-cell junction	34	3.85	-4.9	-3.05
GO:0034045	GO Cellular Components	phagophore assembly site membrane	3	2.56	-4.51	-2.24
GO:0005819	GO Cellular Components	spindle	28	3.17	-4.5	-2.68
GO:0005874	GO Cellular Components	microtubule	30	3.4	-4.49	-2.68
GO:0045121	GO Cellular Components	membrane raft	25	2.83	-4.41	-2.64
GO:0000932	GO Cellular Components	P-body	5	4.27	-4.4	-2.24
GO:0061695	GO Cellular Components	transferase complex, transferring phosphorus-containing groups	21	2.38	-4.27	-2.53
GO:0031253	GO Cellular Components	cell projection membrane	25	2.83	-4.13	-2.42
GO:1904115	GO Cellular Components	axon cytoplasm	4	3.42	-4	-2.02
GO:0019898	GO Cellular Components	extrinsic component of membrane	23	2.6	-3.99	-2.34
GO:0098687	GO Cellular Components	chromosomal region	25	2.83	-3.99	-2.34
ko04550	KEGG Pathway	Signaling pathways regulating pluripotency of stem cells	7	5.98	-5.71	-2.84
ko04140	KEGG Pathway	Autophagy - animal	15	1.7	-4.92	-2.72
ko05224	KEGG Pathway	Breast cancer	16	1.81	-4.91	-2.72
hsa04310	KEGG Pathway	Wnt signaling pathway	6	5.13	-4.52	-2.39
hsa05211	KEGG Pathway	Renal cell carcinoma	3	8.57	-4.14	-2.34
hsa05217	KEGG Pathway	Basal cell carcinoma	8	0.91	-3.52	-1.76
hsa03018	KEGG Pathway	RNA degradation	4	3.42	-3.52	-1.91
ko04015	KEGG Pathway	Rap1 signaling pathway	17	1.93	-3.44	-1.75
hsa04630	KEGG Pathway	Jak-STAT signaling pathway	14	1.59	-3.38	-1.74
hsa05142	KEGG Pathway	Chagas disease (American trypanosomiasis)	11	1.25	-3.27	-1.67
ko04728	KEGG Pathway	Dopaminergic synapse	12	1.36	-3.09	-1.53
hsa04070	KEGG Pathway	Phosphatidylinositol signaling system	10	1.13	-3.03	-1.53
hsa05412	KEGG Pathway	Arrhythmogenic right ventricular cardiomyopathy (ARVC)	8	0.91	-2.73	-1.35
ko05033	KEGG Pathway	Nicotine addiction	4	0.96	-2.54	-1.23
ko04141	KEGG Pathway	Protein processing in endoplasmic reticulum	7	1.97	-2.27	-1.15
hsa05010	KEGG Pathway	Alzheimer’s disease	7	1.97	-2.2	-1.13
M00056	KEGG Pathway	O-glycan biosynthesis, mucin type core	3	0.72	-2.14	-1.1
hsa05222	KEGG Pathway	Small cell lung cancer	5	1.2	-2.08	-1.1

### PPI Network

**Figure 7:**
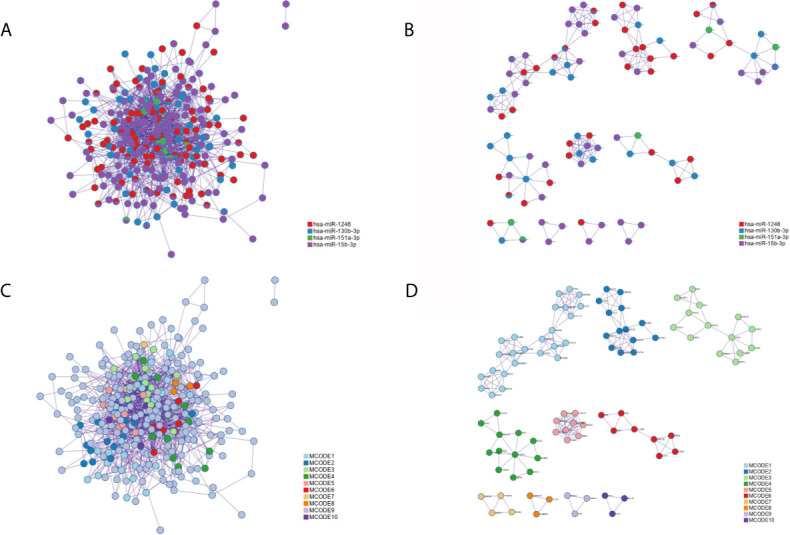
The protein-protein interaction (PPI) network of all common targets. **(A)** Network colored by DEmiRNAs group. **(B)** MCODE subnetworks colored by DEmiRNAs group. **(C)** Network colored by MCODE clusters. **(D)** MCODE subnetworks colored by MCODE clusters.

Applying the STRING and BioGrid data, the PPI network was generated. Meanwhile, each MCODE network was drawn assigned by a unique color (**Figures 7B, D**) or by the matched DEmiRNAs (**Figures 7A, C**). Some genes were located at key nodes, such as TAOK1, CMTM6, SCN3A, WASF3, IGF1, CNOT7, GABRG1 and PRKD1. And the information of these key genes from mcode screening was exhibited in **Table S1**.

### Network Hub Gene Expression Analysis

**Figure 8:**
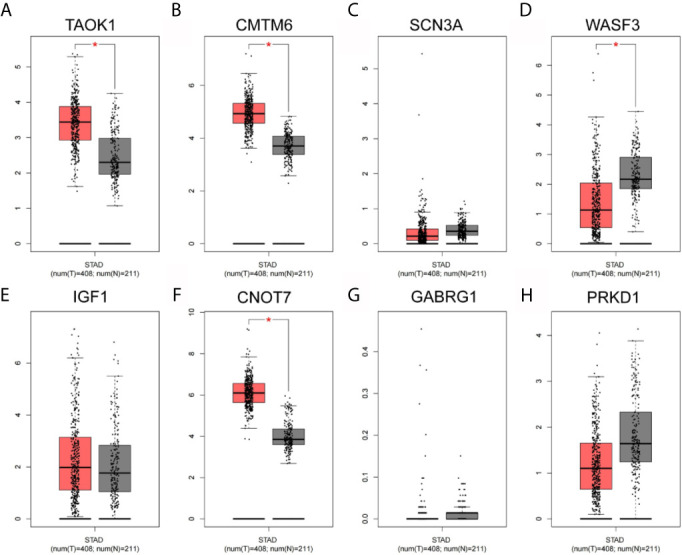
Network hub gene expression analysis. **(A)** TAOK1. **(B)** CMTM6. **(C)** SCN3A. **(D)** WASF3. **(E)** IGF1. **(F)** CNOT7. **(G)** GABRG1. **(H)** PRKD1.

As shown in **Figure 8**, the expression levels of eight key genes screened by MCODE in STAD tissues of TCGA were analyzed. The results showed that the expression of TAOK1, CMTM6 and CNOT7 were significantly lower in the adjacent cancers, while the expression of WASF3 was significantly higher in the adjacent cancers. SCN3A, IGF1, GABRG1 and PRKD1 were not significantly expressed.

## Discussion

Previous bioinformatic studies have identified amounts of GC related miRNAs (3, 5–8). Some circulating serum exosomal miRNAs were proposed as novel biomarkers for GC diagnosis, such as miR-92a-3p, miR-500a-3p, miR-1246, and miR-423-5p (3, 5, 9–12). Especially, some exosomal miRNA can be even used for GC prognosis, e.g., miR-590-5p and miR-196a-1 (3, 13). In this study, we used collected blood and urine samples to analyze the EmiRs associated with GC development. The sequencing results showed four essential circulating exosomal microRNAs: hsa-miR-130b-3p, hsa-miR-151a-3p, hsa-miR-15b-3p and hsa-miR-1246. The targets of these EmiRs and enriched ontology terms should be paid more attention in GC research.

There is a study has reported that miR-130b-3p was upregulated in GC tissues, and miR-130b-3p promoted survival, metastasis and angiogenesis of GC cells as well as enhanced tumor formation and angiogenesis in GC *in vivo* (14). Another one has identified exo-miR-15b-3p/DYNLT1/Caspase-3/Caspase-9 axis do promotes GC development and malignant transformation. It means that serum exo-miR-15b-3p may be a potential GC diagnosis and prognosis biomarker, which can be used in precise targeted GC therapy. Studies *in vitro* revealed that elevated serum miR-1246 was tumor-derived by being packaged into exosomes with the help of ELAVL1 (15). And in a other area, exosomal miR-1246 expressions in serum could differentiate GC patients with TNM stage I from healthy controls and patients with benign diseases (11). Furthermore, there are no correlation between hsa-miR-130b-3p, hsa-miR-151a-3p, hsa-miR-15b-3p and hsa-miR-1246 expression and phenotypic features in the TCGA Stomach database. More clinical studies are to be performed for clarifying the definite roles of above three DEmiRNAs in GC.

A literature search of important hub genes revealed that CKLF like Marvel transmembrane domain 6 (CMTM6) was involved in gene epigenetic regulation and tumorigenesis, and the combined cmtm6 and PD-L1 testing could be used as an indicator to determine the prognosis of patients with gastric cancer (16, 17). Whereas Wiskott Aldrich syndrome protein family member 3 (WASF3) is required for tumor invasion and metastasis. Reports have indicated that WASF3 expression is associated with poor prognosis and is a potential prognostic factor for gastric cancer patients, therefore, targeting WASF3 is a novel potential therapeutic strategy for gastric cancer (18, 19). In addition to this, it has also been documented that mir-218 can inhibit the proliferation, migration and EMT of gastric cancer cells SGC7901 by targeting WASF3 (20). Protein kinase D3 (PRKD3) promotes cancer cell proliferation, growth, migration, and invasion in various tumor types. A growing body of data supports that PRKD3 is a promising therapeutic target for the treatment of cancer (21).

In the PPI network, most of the key nodes were promising candidates that have been fully surveyed, such as TAOK1, CMTM6, SCN3A, WASF3, IGF1, CNOT7, GABRG1 and PRKD1.

Still, this work has some limitations. First, the published studies about hsa-miR-130b-3p, hsa-miR-151a-3p, hsa-miR-15b-3p and hsa-miR-1246 are very few, and known conclusions implied that they are tumor suppressors in all possibility. Again, many targets of our up-regulated DEmiRNAs have been reported to be oncogenic factors or increased in GC (as mentioned above). Their conclusions sharply contradict ours, and the deep reasons need to be further explored. Second, we failed to acquire any common DE-miR in blood and urine, which may be due to that only 3 or 7 samples were included in each group. Theoretically, there may be some enriched circulating exosomal miRNAs in both blood and urine, and more samples should be accumulated in future.

## Conclusions

In conclusion, this study provides an integrative analysis of expression profile of key circulating exosomal microRNAs. Four key miRNAs were found: hsa-miR-130b-3p, hsa-miR-151a-3p, hsa-miR-15b-3p and hsa-miR-1246. These risk exosomal microRNAs, as well as the corresponding interaction network or enrichments based on their targets (such as TAOK1, CMTM6, SCN3A, WASF3, IGF1, CNOT7, GABRG1, PRKD1) may provide a better understanding of the molecular mechanisms in the GC development.

## Data Availability Statement

The datasets presented in this study can be found in online repositories. The names of the repository/repositories and accession number(s) can be found in the article/**Supplementary Material**.

## Ethics Statement

The studies involving human participants were reviewed and approved by The Ethics Committee of Renji Hospital (Shanghai, China). The patients/participants provided their written informed consent to participate in this study.

## Author Contributions 

Conception and design: XQ. Administrative support: DC. Provision of study materials or patients: HW. Collection and assembly of data: FX. Data analysis and interpretation: XQ. Manuscript writing: All authors. All authors contributed to the article and approved the submitted version.

## Funding

This work was supported by Key Basic Research Program of China (No.2017YFA0205304), Nature Scientific Foundation of China (No.820201080, 81602184, SKLPBS1827), and Medical Engineering Cross Project of Shanghai Jiao Tong University (YG2016ZD10, ZH2018QNA51, ZH2018QNA28). This work was also supported by “the Belt and Road” young scientist exchange program of the Science and Technology Commission of Shanghai (Grant No. 18410741600).

## Conflict of Interest

The authors declare that the research was conducted in the absence of any commercial or financial relationships that could be construed as a potential conflict of interest.
