# Supplementary material for: Identification of Key Circulating Exosomal microRNAs in Gastric Cancer
Source: Front Oncol. 2021 Jul 16;11:693360. doi: 10.3389/fonc.2021.693360 (PMC8323470; doi:10.3389/fonc.2021.693360)
Supplement: Supplementary file 1 [file Table_1.docx]

### **Table S1　The information of key genes from MCODE.**

| Cluster | Score | Type | Gene | Symbol |
| --- | --- | --- | --- | --- |
| 1 | 2.96 | Clustered | 51552 | RAB14 |
| 1 | 2.96 | Clustered | 5932 | RBBP8 |
| 1 | 2.96 | Clustered | 950 | SCARB2 |
| 1 | 2.96 | Clustered | 5286 | PIK3C2A |
| 1 | 2.96 | Clustered | 201475 | RAB12 |
| 1 | 2.96 | Clustered | 948 | CD36 |
| 1 | 2.96 | Clustered | 2072 | ERCC4 |
| 1 | 2.96 | Clustered | 8322 | FZD4 |
| 1 | 2.96 | Clustered | 27314 | RAB30 |
| 1 | 2.96 | Clustered | 5912 | RAP2B |
| 1 | 2.96 | Clustered | 7486 | WRN |
| 1 | 2.96 | Seed | 54918 | CMTM6 |
| 1 | 2.96 | Clustered | 961 | CD47 |
| 1 | 2.96 | Clustered | 5865 | RAB3B |
| 1 | 2.96 | Clustered | 57403 | RAB22A |
| 1 | 2.96 | Clustered | 1080 | CFTR |
| 1 | 2.96 | Clustered | 30011 | SH3KBP1 |
| 1 | 2.96 | Clustered | 23250 | ATP11A |
| 1 | 2.96 | Clustered | 683 | BST1 |
| 1 | 2.96 | Clustered | 5795 | PTPRJ |
| 1 | 2.96 | Clustered | 672 | BRCA1 |
| 1 | 2.96 | Clustered | 51426 | POLK |
| 1 | 2.96 | Clustered | 51209 | RAB9B |
| 1 | 2.96 | Clustered | 5985 | RFC5 |
| 1 | 2.96 | Clustered | 1601 | DAB2 |
| 2 | 2.571428571 | Clustered | 2259 | FGF14 |
| 2 | 2.571428571 | Clustered | 23236 | PLCB1 |
| 2 | 2.571428571 | Clustered | 6323 | SCN1A |
| 2 | 2.571428571 | Seed | 6328 | SCN3A |
| 2 | 2.571428571 | Clustered | 6335 | SCN9A |
| 2 | 2.571428571 | Clustered | 2256 | FGF11 |
| 2 | 2.571428571 | Clustered | 2775 | GNAO1 |
| 2 | 2.571428571 | Clustered | 805 | CALM2 |
| 2 | 2.571428571 | Clustered | 1739 | DLG1 |
| 2 | 2.571428571 | Clustered | 793 | CALB1 |
| 2 | 2.571428571 | Clustered | 2903 | GRIN2A |
| 2 | 2.571428571 | Clustered | 2891 | GRIA2 |
| 2 | 2.571428571 | Clustered | 1740 | DLG2 |
| 2 | 2.571428571 | Clustered | 2890 | GRIA1 |
| 3 | 1.5 | Clustered | 6434 | TRA2B |
| 3 | 1.5 | Clustered | 167227 | DCP2 |
| 3 | 1.5 | Clustered | 22976 | PAXIP1 |
| 3 | 1.5 | Clustered | 23468 | CBX5 |
| 3 | 1.5 | Clustered | 10006 | ABI1 |
| 3 | 1.5 | Clustered | 57472 | CNOT6 |
| 3 | 1.5 | Seed | 10810 | WASF3 |
| 3 | 1.5 | Clustered | 25 | ABL1 |
| 3 | 1.5 | Clustered | 84617 | TUBB6 |
| 3 | 1.5 | Clustered | 5160 | PDHA1 |
| 3 | 1.5 | Clustered | 27161 | AGO2 |
| 3 | 1.5 | Clustered | 2673 | GFPT1 |
| 3 | 1.5 | Clustered | 6733 | SRPK2 |
| 3 | 1.5 | Clustered | 1656 | DDX6 |
| 4 | 1.416666667 | Clustered | 4254 | KITLG |
| 4 | 1.416666667 | Clustered | 2280 | FKBP1A |
| 4 | 1.416666667 | Clustered | 2065 | ERBB3 |
| 4 | 1.416666667 | Clustered | 79056 | PRRG4 |
| 4 | 1.416666667 | Clustered | 9706 | ULK2 |
| 4 | 1.416666667 | Clustered | 3667 | IRS1 |
| 4 | 1.416666667 | Clustered | 10019 | SH2B3 |
| 4 | 1.416666667 | Clustered | 83737 | ITCH |
| 4 | 1.416666667 | Clustered | 5291 | PIK3CB |
| 4 | 1.416666667 | Clustered | 2069 | EREG |
| 4 | 1.416666667 | Clustered | 22863 | ATG14 |
| 4 | 1.416666667 | Clustered | 145957 | NRG4 |
| 5 | 4 | Clustered | 23279 | NUP160 |
| 5 | 4 | Clustered | 23332 | CLASP1 |
| 5 | 4 | Clustered | 5516 | PPP2CB |
| 5 | 4 | Clustered | 5885 | RAD21 |
| 5 | 4 | Clustered | 81565 | NDEL1 |
| 5 | 4 | Clustered | 5048 | PAFAH1B1 |
| 5 | 4 | Clustered | 6249 | CLIP1 |
| 5 | 4 | Clustered | 8655 | DYNLL1 |
| 5 | 4 | Seed | 57551 | TAOK1 |
| 6 | 1.5 | Clustered | 2066 | ERBB4 |
| 6 | 1.5 | Clustered | 4040 | LRP6 |
| 6 | 1.5 | Clustered | 1398 | CRK |
| 6 | 1.5 | Seed | 3479 | IGF1 |
| 6 | 1.5 | Clustered | 8323 | FZD6 |
| 6 | 1.5 | Clustered | 7855 | FZD5 |
| 6 | 1.5 | Clustered | 7482 | WNT2B |
| 6 | 1.5 | Clustered | 2241 | FER |
| 7 | 1.25 | Clustered | 149041 | RC3H1 |
| 7 | 1.25 | Clustered | 1654 | DDX3X |
| 7 | 1.25 | Clustered | 253943 | YTHDF3 |
| 7 | 1.25 | Seed | 29883 | CNOT7 |
| 8 | 1 | Seed | 2565 | GABRG1 |
| 8 | 1 | Clustered | 25932 | CLIC4 |
| 8 | 1 | Clustered | 2561 | GABRB2 |
| 9 | 1 | Clustered | 5581 | PRKCE |
| 9 | 1 | Clustered | 4067 | LYN |
| 9 | 1 | Seed | 5587 | PRKD1 |
| 10 | 1 | Clustered | 4299 | AFF1 |
| 10 | 1 | Clustered | 22936 | ELL2 |
| 10 | 1 | Clustered | 4300 | MLLT3 |
